# Health and Religion

**Published:** 1875-07

**Authors:** George H. Smyth

**Affiliations:** Chaplain, House of Refuge, Randall’s Island


### HEALTH AND RELIGION.

(COMMUNICATED.)

No men deserve the world's gratitude more than those who are laboring to teach the people how to take care of their *bodies ;* not even the men who are laboring to teach them how to take care of their *souls.*

I say this as a minister of the gospel, believing that the proper regulation of the body involves the highest interest of the soul. I shall not wait to argue this point. Suffice it to say that by far the largest portion of the Old Testament is taken up with the care of the former, while the New Testament shows plainly that the great Physician of both soul and body always cared for the latter—feeding, healing and helping it whenever necessity required. Through men's bodies he reached their souls. The morbid appetites, passions and desires of the body are the fruitful sources of much of man's sin and sorrow. Hence the Apostle's prayer for the Thessalonian Christians—" The very God of peace sanctify you wholly; and I pray God your whole spirit and soul and BODY, be preserved blameless unto the coming of our Lord Jesus Christ."

Many of the dissensions, divisions and heresies that have vexed the Christian Church owe their origin quite as much to a torpid *stomach* as to a depraved heart. Many failures in business, family quarrels and unhappy deaths might be traced to the same source.

I had a class-mate at boarding-school one of the most genial, joyous and happy fellows I ever met, and one of the most beautiful and devoted Christians. He got into the habit of " bolting" his food, seldom taking longer than five minutes to any one meal—a feat in which many of the other boys outrivaled him. On one occasion he told me his bowels had not been moved for eight days. I said " you will kill yourself." "Oh no, I am just as well as ever, and eat heartily every meal," was his reply. Soon after he required the daily attendance of a physician for several weeks. A confirmed dyspepsia was the result. He grew morbid, unhappy and disagreeable. We went to different colleges, but met five years after at the Theological Seminary. He had hardly a friend among the students. I kn[illegible] the cause of the wonderful chang[illegible] that had come over the once bright, happy, lovable boy, and which had made him a withered, sallow, irritable man. I loved him still, pitied him, humored him in his whims and cheered many of his melancholy moments.

He was talented, a thorough student, a profound thinker and a most conscientious christian, but owing to his chronic hypochondria, his ministry has been comparatively a failure.

I visited a member of my church once who was suffering from constipation. I warned him and his friends of the danger I saw approaching. It came ; first insanity and then suicide.

"Father forgive them for they know not what they do," and give great success to those who are endeavoring to teach them, for every day confirms the sad truth of the Scripture—"My people are destroyed for lack of knowledge."

I fully appreciate the great work which your valuable magazine is doing, and trust you will go forward as earnestly as heretofore, until Physiology, instead of German, is taught in our public schools. With proper objects and models, I see no reason why even the infant classes may not be taught a knowledge of themselves, and thus be saved from a multitude of errors which are now chargable solely to the universal ignorance of our race.

GEORGE H. SMYTH, Chaplain,
House of Refuge, Randall's Island.

SUNDAY CHANGES.—Sunday is a [illegible]ad day on which to change from heavy winter clothing to the lighter garb of summer. Of course we recognize no *spring* garments, for it is the dictate of prudence, in New York, to hold to the dress and under clothing of winter until the first or middle of June. But when we do don our light apparel let it not be on a Sunday. If we do we are quite sure to sit in a draft while in church, or to feel a slight chillness when taking our afternoon nap at home, and then before we know it, we have taken cold. It is much safer to make our changes from heavy to light clothes on a day when we are active about our business, for we are then less likely to be exposed to drafts, and our usual exercise will protect us from chill and cold.

---

Frank sincerity, though no invited guest, is free to all, and brings his welcome with him.

